# The financial protection effect of Ghana National Health Insurance Scheme: evidence from a study in two rural districts

**DOI:** 10.1186/1475-9276-10-4

**Published:** 2011-01-19

**Authors:** Ha TH Nguyen, Yogesh Rajkotia, Hong Wang

**Affiliations:** 1 International Health Division, Abt Associates Inc., 4550 Montgomery Avenue, Suite 800 North, Bethesda, MD 20814, USA; 2 Bureau for Global Health, United States Agency for International Development, 1300 Pennsylvania Avenue, Washington DC 20523, USA

## Abstract

**Background:**

One of the key functions of health insurance is to provide financial protection against high costs of health care, yet evidence of such protection from developing countries has been inconsistent. The current study uses the case of Ghana to contribute to the evidence pool about insurance's financial protection effects. It evaluates the impact of the country's National Health Insurance Scheme on households' out-of-pocket spending and catastrophic health expenditure.

**Methods:**

We use data from a household survey conducted in two rural districts, Nkoranza and Offinso, in 2007, two years after the initiation of the Ghana National Health Insurance Scheme. To address the skewness of health expenditure data, the absolute amount of out-of-pocket spending is estimated using a two-part model. We also conduct a probit estimate of the likelihood of catastrophic health expenditures, defined at different thresholds relative to household income and non-food consumption expenditure. The analysis controls for chronic and self-assessed health conditions, which typically drive adverse selection in insurance.

**Results:**

At the time of the survey, insurance coverage was 35 percent. Although the benefit package of insurance is generous, insured people still incurred out-of-pocket payment for care from informal sources and for uncovered drugs and tests at health facilities. Nevertheless, they paid significantly less than the uninsured. Insurance has been shown to have a protective effect against the financial burden of health care, reducing significantly the likelihood of incurring catastrophic payment. The effect is particularly remarkable among the poorest quintile of the sample.

**Conclusions:**

Findings from this study confirm the positive financial protection effect of health insurance in Ghana. The effect is stronger among the poor group than among general population. The results are encouraging for many low income countries who are considering a similar policy to expand social health insurance. Ghana's experience also shows that instituting insurance by itself is not adequate to remove fully the out-of-pocket payment for health. Further works are needed to address the supply side's incentives and quality of care, so that the insured can enjoy the full benefits of insurance.

## Background

The World Bank [1] posited that for any health financing coverage, there are generally three interrelated and separate dimensions: the breadth (number of people covered), the depth (the extent of services covered), and the resulting impacts on health outcomes and financial protection against large out-of-pocket (OOP) expenditures. While documenting the first two dimensions is rather straightforward, evaluating the impacts of health financing interventions is not an easy undertaking. To date, the evidence of impact on health outcomes is scarce and that on financial protection does not support a unanimous conclusion. Regarding the latter, while many studies show that insurance helped reducing the OOP health spending [2,3], others found that, by altering the utilization patterns, insurance could lead to increased OOP expenditure [4] and higher likelihood of catastrophic payment [5,6].

The current study analyzes the case of Ghana National Health Insurance Scheme (NHIS) and contributes to evidence on the financial protection provided by health coverage. Using data from two rural districts of Ghana, we evaluate the potential protective impact of NHIS on the financial burden of health care as measured in the total OOP amount spent on health and the probability that an individual encounters catastrophic health payment. While several studies have looked at the implementation aspect of NHIS [7], its equity implications in terms membership composition [8], and its impact on service utilization [9], to our knowledge there has been no in-depth assessment of the NHIS's financial protection effect. Yet this is the principal policy objective for the NHIS, as expressed in the Ghanaian government's national health insurance policy framework:

"Within the next five years, every resident of Ghana shall belong to a health insurance scheme that adequately covers him or her against the need to pay out of pocket at the point of service use in order to obtain access to a defined package of acceptable quality of health service."

[10, cited in 7]

The next section provides an overview of Ghana's health financing and the NHIS. This is followed by a description of the methods used to address the study's research questions. We next present our results and discuss the implications of the study's findings. The final section concludes.

### Overview of Ghana's Health Financing and the NHIS

With a gross domestic product per capita of US$ 306 in 2007, Ghana is a low income country in the sub-Saharan Africa region. The country compares positively with peers in sub-Saharan Africa in a number of important health indicators, such as maternal and child mortality, fertility rate, and HIV/AIDS prevalence among adults ages 15-49 [11]. However, Ghana also pays more for health as percentage of gross domestic product (6.2 percent in Ghana compared to the sub-Saharan Africa's average of 5.3 percent in 2006) [11].

Similar to many countries coming out of the colonial regime, after independence in 1957, the Ghanaian government adopted a tax-based health financing system in which the government paid for services in the public sector. By the early 1970s, general tax revenue in Ghana, with its stagnating economy, could not support a tax-based health financing system. Ghana started to introduce nominal user fees in the public sector, which in 1985 were raised significantly with the aim to recover at least 15% of recurrent expenditure [7]. This user fee system, known as "cash and carry," was shown to leave negative consequences in access to health services, especially by the poor people [12,13]. To offset the adverse effects of user fees, the government introduced exemption policy for children, pregnant women, the elderly, the extreme indigents, and persons suffering from certain communicable diseases. In practice the exemption did not work well, and many of those who should have been exempted were not [14]. A number of adverse effects of the "cash and carry" system have been reported, including long delays in seeking health services when ill and incomplete prescription purchases [15]. In the beginning of the years 2000s, the share of household OOP payment in total health expenditure in Ghana was considerably higher than the regional average for the sub-Saharan Africa (50 percent versus 39 percent respectively in 2006) [11].

The establishment of the NHIS in 2003 is a major step by the Ghanaian government to address the perverse problems of the "cash and carry" system. NHIS was fueled partly by the relative success of the numerous mutual health organizations (MHOs), which existed in 67 out of 138 districts in Ghana with very diverse management structures and benefit packages [16]. However, NHIS marks an important departure from the MHO prepayment mechanism. It mandated the establishment of an MHO in every district and contracted existing private health insurance schemes. Although the administration of NHIS remains decentralized at the district level, its financing is centralized and the benefit package is standardized across the whole country. NHIS's benefit package covers a wide range of outpatient services with associated drugs and lab tests, inpatient care, treatment of cervical and breast cancers, basic oral health services, eye care, maternal care, and all emergency conditions. No coinsurance, copayment, or deductible is required at the point of service [17]. All Ghanaians, from both the formal and informal sectors, are in principle required to enroll. Public health facilities in the country are automatically accredited to contract with the NHIS, and private health facilities can apply for accreditation. It is estimated that in 2008, 479 private facilities were accredited while a larger number remained without accreditation [18].

Funding for NHIS comes mainly from a National Health Insurance sales tax of 2.5 percent, a transfer of 2.5 percent of formal sector contributions to the Social Security and Pension Scheme Fund, and from member contributions. In the beginning years of the scheme, 70 - 75 percent of total insurance revenue came from tax revenue, 20-25 percent from formal sector contribution, and only about 5 percent from contribution of members in the informal sector [19]. Premium is exempted for several member categories, including Social Security and National Insurance Trust Fund pensioners, children under age 18, adults above age 70, and the indigents [19]. As of 2008, the NHIS provided 41 percent of the total public resource envelope and about 45 percent of the Ghanaian population were NHIS cardholders [18]. This coverage level actually outperforms the targets set in the policy document, which aims to insure 30-40 percent of the population by 2010 [16].

Early experience in the implementation of NHIS reveals a number of problems. Despite the explicit policy to exempt premium for the indigents, poor people were much less likely to be insured than the better off [8,9]. There has been serious delay in issuing cards for members, as well as in reimbursing the providers. Delay in provider reimbursement and inadequate supply side incentives are deemed responsible for the lower quality of care received by insured patients compared to non-insured, paying patients. Anecdotal examples of poor quality of care include provider discrimination against insured patients, long waiting time, low likelihood of being seen by a doctor and of receiving all drugs prescribed [18]. In particular, it has been reported that providers commonly solicited informal payments from the patients by charging for services out-of-hours, asking patients to pay for drugs, which are said not to be in stock, and asking patients to pay for "better" drugs, said to be not provided under the NHIS [18].

To date the impact of NHIS on health service utilization and OOP expenditure has not been rigorously evaluated at the national level. Several studies conducted in a small number of districts suggested that NHIS has encouraged utilization of curative health services [9,20]. None of these studies, however, successfully dealt with the issue of adverse selection of NHIS membership.

## Methods

### Measures of the Financial Protection Effects of Health Insurance

One of the key values of health insurance is to help people deal with the unpredictability of illness and medical spending [21]. It is well known that health expenditure is extremely variable and skewed, with a disproportionately large amount usually concentrated in a small portion of the population. For example, data from the United States in 1987 show that the top 10 percent of users account for nearly 75 percent of total medical spending [22]. Without a proper risk pooling mechanism, households that are unfortunate enough to have catastrophic illnesses will find it very difficult to cover the medical bills. The financial consequences of ill health can be especially devastating for poor people, who already struggle to cover the basic daily needs such as food and shelter. A recent analysis of 15 African countries revealed that in most of these countries, around 30 percent of all households financed OOP health expenditure by borrowing and selling assets [23]. Health insurance is designed to reduce the amount that households have to pay OOP when they use medical services.

Looking at the absolute amount of OOP payment changed as the result of having health insurance is not adequate to judge its effect on the financial burden of health care. Low OOP payment may just derive from the fact that insurance does not trigger any increase in utilization; hence no additional payment is needed. On the other hand, high OOP payment may not necessarily be bad if it buys substantial improvements in quality and/or quantity of services and if the households can afford to pay [6]. Thus, the issue of financial burden is more directly related to capacity to pay than to the absolute amount.

Along this line of argument, Wagstaff and van Doorslaer [24] defined a concept of "catastrophic" health expenditure - expenditure that exceeds some pre-specified fraction of household income. The idea is that health spending comes at the expense of other household consumption expenditure, and health spending should not exceed a certain threshold z, so that households have at least (1-z) of their income to spend on other needs. The catastrophic expenditure indicator is a commonly accepted tool for measuring the financial burden of health care relative to the household capacity to pay. The concept has been employed widely to portray the health financing profile and to evaluate the impact of health insurance in many countries [5,25-27]. Although one may argue that z should vary by income level, because rich people may afford to spend higher portion of their income on health compared to the poor, no theoretical or empirical work has been conducted to date to address this issue.

Following the practice of the existing literature, we adopt two measures of the financial protection effects of insurance in this study. They include the absolute amount of OOP expenditure and the likelihood of high or catastrophic expenditure.

### Model Specifications

A generic model for estimating the impact of health insurance for an individual *i *with the outcome of interest *Y *can be estimated as followed:

$${\text{Y}}_{\iota }=\text{F}({\text{HI}}_{\iota },\;{\text{X}}_{\iota },\;{\text{Z}}_{\iota },\varepsilon_{\iota })$$

where the F function in health service research typically takes the form of linear, probit, logit, or poisson depending on the distribution of the outcome variable, HI denotes insurance status, X is a vector of observable characteristics, Z is a vector of characteristics unobservable to the researchers, and ε is the stochastic term, usually assumed to be normally distributed. Note that included in X and Z could be values shared by all household members.

Because Z is unobservable, it is included in the error term. The estimate of the health insurance (HI) effect on Y will be biased if Z contains at least one element that is systematically correlated with both insurance status and the outcome of interest (HI is endogenous). For example, if Y is a measure of health service utilization, typical candidates for such an element of Z would be the individual's risk aversion or poor health condition, which are likely to be positively correlated with both insurance and Y. In such a case, failure to control for this interfering effect of the omitted variables will lead to an overestimation of the insurance effect. Unfortunately, while tools for dealing with this omitted variable bias are widely known (such as randomized controlled study, individual fixed effects model, or instrumental variable), in reality these tools often are not readily available.

In this study, we obtained information on the individual chronic health condition and whether the study respondents assessed their health status as "bad." While ultimately there will be other confounding factors that we have not controlled for, poor health status is a commonly cited reason for adverse selection in the insurance literature [21]. Thus being able to account for poor health status helps improve the validity of the study findings. More importantly, for the type of outcome studied (health expenditure), failure to control for the omitted variable bias will likely lead to a lower bound estimate of the insurance effect, provided that the signs of correlation between such omitted variable with both insurance status and the outcome are similar. This ability to bound the insurance effect is important - if we find some significant result, we know the true effect will be significant.

### Data and Variables

#### Data

The data for this study come from a household survey in two districts of Ghana conducted in September-October 2007. The survey was funded by the United States Agency for International Development and conducted by the Health Systems 20/20 Project, led by Abt Associates Inc., in collaboration with the Research and Development Division of the Ghana Health Services. The two districts are Nkoranza and Offinso, both predominantly agricultural and relatively poor. Nkoranza is one of the districts in the country where a MHO operated before the NHIS; Offinso had no prior experience with community-based financing.

Table 1 provides a description of the two study districts. The population in the two districts, respectively, is 130 and 140 thousand, slightly less than the average for this country of 21 million and 138 districts. As reflected by the proportion of rural population and the administrative classification of "deprived" district, Nkoranza appears to be more disadvantaged than Offinso. Correspondingly, insurance registration fees and premiums were set higher in Offinso than in Nkoranza. According to NHIS administrative data, by 2007, 36 percent of Offinso's population had registered for NHIS; the corresponding figure for Nkoranza was 45 percent.

**Table 1:** Description of the Study Sites

	Nkoranza district	Offinso district
Demographic and health services		
Total population	128,960	138,676
Rural population (%)	69	58
Economic status (administratively classified)	"deprived"	"less deprived"
Number of hospitals	1	2
Number of public health centers	12	7
Number of private and mission clinics	1	6
NHIS administrative data		
% population registered	45	36
Registration fee (Cedi)	20,000-30,000	20,000-50,000
Annual premium (Cedi)	80,000	150,000
Benefit package	95% of disease conditions. Various services, drugs, and tests belonging to outpatient services, inpatient care, oral health, maternity care, and emergency care.	

*Sources*: Ghana districts http://www.ghanadistricts.com, Health System 20/20 Project and Ghana Health Services (2009).

*Note*: Figures on total and rural populations come from 2000 census. All other values are current as of 2007.

Cedi is old Ghana Cedi 2007 current value; exchange rate: 1 USD = 9,302 Cedi (2007)

The household survey applied a two-stage cluster sampling approach. In the first stage, municipalities in the urban and rural areas (towns and villages) were selected based on population size and geographical dispersion. In the second stage, households were randomly selected from each municipality. Insured people were oversampled to increase power and sampling weights were used to adjust for this fact. A total of 2,500 households were surveyed, giving a final sample of 5,879 individuals in Offinso and 5,738 in Nkoranza.

#### Variables

The household survey collected detailed information on the insurance status of all household members. It recorded any incidence of illness and injury over the two weeks preceding the survey, antenatal care, delivery, and hospitalization over the preceding 12 months, as well as the associated OOP payment for such events. From this information, we computed a variable for annual OOP health expenditure (two-week expenditure multiplies by 26, plus 12-month expenditure on hospitalization and delivery care). Note that using two-week expenditure on illness and injury to extrapolate to a 12-month figure could be problematic due to seasonality of illness and related health care seeking. However, because the field survey only last for more than a month, any potential seasonality problem should be the same for NHIS members and non-members. The derived OOP expenditure variable covers all sources, informal and formal care, from both the government and private sectors. Note that this is expenditure associated with specific health problems only; it does not include preventive care. The survey also collected information on ownership of land, assets (TV, fridge, telephone, bicycle, motorbike, and car), and living conditions (whether there is electricity, floor type, type of water, and fuel used for cooking; owning a house, having farmland, and number of rooms in the house). From this information, a wealth quintile variable was constructed using the principle component method.

In this study, outcomes of interest include annual OOP health expenditure on curative care and measures of catastrophic health payment. For the latter, a practical difficulty is that the survey did not collect information on household income or consumption expenditure to be used for comparison. We therefore use an indirect measure, comparing the health expenditure of individuals in each wealth quintile in the sample with the average income and non-food consumption expenditure of the corresponding quintile obtained from Ghana's latest Living Standard Survey (2005-2006) [28]. In doing that, we adjusted quintile specific income and non-food consumption expenditure from the Ghana 2005-2006 Living Standard Survey to 2007 values, taking into account the inflation rate and real growth in the country. A closely related approach was adopted by Wagstaff and Lindelow [6] who defined health spending as "high" if it exceeded 5 percent of average income in their study sample. Following the practice in the existing literature, we constructed four indicators of catastrophic OOP expenditure, i.e., expenditure that exceeds one of the following four thresholds: 5 percent of quintile specific household income per capita, 10 percent of income, 10 percent of non-food consumption expenditure, and 20 percent of non-food consumption expenditure.

The key independent variable of interest is whether an individual had NHIS at the time of survey. In addition to education, occupation, and gender of the household head, the covariates include individual self-reported chronic health conditions and self-assessed health status, gender, ethnic Akan, household size, assets and living conditions, and whether at least one member of the household joined a local solidarity scheme. These covariates are included because they can potentially confound the insurance - OOP expenditure relationship. For example, richer households may be more likely to afford health insurance while paying more for health services at the same time. Therefore, not controlling for wealth status may lead to overstating the amount that insured people pay for health services. The last variable, membership in a local solidarity scheme, serves as an indicator of social capital, which could potentially be correlated with insurance status and health service outcomes.

#### Estimation methods

In this paper, binary outcomes will be estimated with a probit model. While both probit and logit are typically used for binary outcome, the former is preferred because it produces estimates for marginal effect, which shows the difference in the probability of having catastrophic payment between the insured and the uninsured. For OOP expenditure, a linear model is not suitable because data are highly skewed with a large number of zeros. Taking log of expenditure data is also not preferred because the large number of observations with zero expenditure will be dropped out. Following the common practice in health service research, we employ a two-part model, which adds flexibility by modeling separate processes to explain the probability of positive expenditure and the average OOP amount [29]. In the first part, the probability of a positive expenditure is estimated with a probit model and, in the second part, a log linear model is used for non-zero expenditure. The combined marginal effect of health insurance is estimated according to Dow and Norton [30] with a Stata program written by Norton [31].

## Results

### Descriptive Statistics

Table 2 presents the breakdown summary of OOP health expenditure by insurance status for the study sample. The sample consists of 4,899 people having NHIS status (members) and 6,718 people without NHIS status (non-members), yielding a weighted insurance rate of 35 percent. This rate is lower than the administrative record of the NHIS shown in Table 1. One reason for this discrepancy could be that members have to wait for six months after registration to begin accessing services, and many received their NHIS card only after a delay. Therefore, the registration information does not give a precise measure of the actual number of cardholders [18]. Because 99 percent of all insured people in the sample have NHIS, we ignore other types of insurance. For simplicity, individuals with NHIS status will be called "insured" in this analysis.

**Table 2:** Breakdown of OOP Expenditure during 12 Months Preceding the Survey by Insurance Status (Cedi)

Expenditure breakdown	NHIS nonmembers (N = 6,718)		NHIS members (N = 4,899)	
	Mean	Standard deviation	Mean	Standard deviation
Acute illnesses and injuries				
Informal care	2,839	37,902	4,913	85,028
Consultation fee	3,854	130,912	346	32,528
Lab expenses	1,354	57,391	1,036	42,923
Other expenses	210	9,760	989	50,711
Unofficial payment to providers	174	6,682	472	16,640
Drugs purchased at facility	6,500	103,561	2,709	79,055
Drugs purchased outside facility	2,348	83,133	3,743	90,928
Antenatal care and delivery	6,442	85,419	4,475	82,859
Surgery and hospitalization	6,121	95,147	2,819	79,136
Total	29,843	278,617	21,503	265,705

*Note*: Cedi is old Ghana Cedi, 2007 current value; exchange rate: 1 USD = 9,302 Cedi (2007)

As shown in Table 2, on average, an insured person paid 21 thousand Cedi over the 12 months preceding the survey and an uninsured person paid nearly 30 thousand Cedi (we report old Cedi used before the redenomination of currency in July 2007; exchange rate: US$1 = Cedi 9,302). Thus, despite the generous benefit package, insured people still incurred an OOP expense roughly 72 percent of that paid by uninsured people.

Some interesting observations emerge from Table 2. First, insured people still report paying for items that should be covered by insurance, such as consultation fees, lab expenses, and drugs purchased at the facility (see description of benefit package in Table 1). This pattern suggests that the NHIS does not fully shield its members from OOP payment at the point of service. Second, compared with the uninsured, insured people pay more for items not covered by insurance, such as informal care, unofficial payment to providers, and purchase of drugs outside the facility. The higher amount spent on informal care among the insured suggests that insured people may indeed sicker than the uninsured. On the other hand, the fact that insured people pay more for drugs purchased outside facility (hence not covered by insurance) and for "other expenses" provides evidence supporting the earlier observation that quality of care may be a problem with insured services. The pooled average of OOP expenditure among the insured and uninsured is 26,922 Cedi (table not shown), rather low compared with the country mean annual per capita income of 3,970,000 Cedi reported in the Ghana Living Standard Survey 2005-2006 (0.68 percent). This figure also is rather low compared with that of other developing countries, especially given that insurance coverage was 35 percent at the time. However, it is important to note that this OOP amount only includes expenditures associated with specific illness, hospitalization, antenatal care, or delivery. Hence it does not capture expenditures associated with preventive care and purchase of drugs for health maintenance.

Table 3 presents the summary statistics of relevant variables. For completeness, it also shows the total OOP expenditure reported in Table 2. As Table 3 shows, only 3-4 percent of the study sample actually incurred any positive OOP payment for health services. The incidence of high and catastrophic expenditure ranges from 0.9 percent to 2.8 percent depending on the indicators. Still, the pattern is very consistent; the incidence of high and catastrophic OOP expenditures is noticeably lower among the insured for all measures, suggesting that the NHIS did have some protective effects. Xu et al. [25], using Ghana 1998-1999 Living Standard Survey data and 40 percent of non-food consumption threshold, estimated a catastrophic incidence rate of 1.3 percent. For the purpose of checking comparability, we also computed a catastrophic expenditure variable based on Xu's et al. threshold using our method (i.e., employing quintile-specific income and expenditure data from Ghana Living Standard Survey 2005-2006 as denominators). The incidence rate obtained is 0.95 percent, which is reasonable given that our estimates and those of Xu et al. used data that were 8-9 years apart. Still, our figures could be slightly underestimated because the OOP variable does not cover all expenses, as mentioned above.

**Table 3:** Summary Statistics of the Study Sample

Variables	NHIS nonmembers (N = 6,718)		NHIS members (N = 4,899)	
	Mean	s.d.	Mean	s.d.
OOP health expenditure (old Cedi)	29,843	278,617	21,503	265,705
Had positive OOP expenditure	0.042	0.202	0.032	0.177
Health expenditure > = 10% non-food consumption expenditure per capita	0.026	0.158	0.015	0.120
Health expenditure > = 20% non-food consumption expenditure per capita	0.020	0.140	0.009	0.094
Health expenditure > = 10% income per capita	0.021	0.143	0.012	0.108
Health expenditure > = 5% income per capita	0.028	0.164	0.015	0.124
Chronic health condition	0.028	0.164	0.063	0.242
Bad health (self-assessed)	0.014	0.119	0.023	0.149
Male	0.485	0.500	0.444	0.497
Age	22.553	19.202	26.088	22.775
Ethnic Akan	0.672	0.470	0.815	0.389
One hh member in solidarity scheme	0.098	0.297	0.147	0.354
Female-headed household	0.299	0.458	0.347	0.476
Hh head has no education	0.364	0.481	0.291	0.454
Hh head education: primary	0.563	0.496	0.579	0.494
Hh head education: > = secondary	0.073	0.260	0.130	0.337
Hh head not working	0.093	0.290	0.091	0.288
Hh head is farmer/fisherman	0.710	0.454	0.644	0.479
Hh head is government worker	0.039	0.193	0.066	0.248
Hh head is artisan	0.134	0.340	0.171	0.377
Hh head is trader	0.024	0.154	0.028	0.165
Quintile 1 (poorest)	0.275	0.446	0.115	0.319
Quintile 2	0.223	0.416	0.174	0.379
Quintile 3	0.171	0.377	0.210	0.407
Quintile 4	0.184	0.387	0.213	0.409
Quintile 5	0.147	0.354	0.289	0.453
Household size	6.317	3.066	5.932	2.791
Urban	0.116	0.320	0.130	0.336

*Note*: The statistics are adjusted for sampling weight.

Hh=household

Cedi is old Ghana Cedi, 2007 current value; exchange rate: 1 USD = 9,302 Cedi (2007)

Table 3 also shows that the proportions of insured people with reported chronic health conditions and bad health status were twice as large as those among the uninsured, suggesting that adverse selection exists. However, health status is not the only factor relating to insurance. As shown, insured people are more likely to be female, from ethnic Akan, and living in smaller, female-headed households and in households with at least one member joining a local solidarity scheme. There is also a very strong socioeconomic gradient of insurance, with coverage being systematically higher among the urban, wealthy, and highly educated. This differential coverage of NHIS among socioeconomic groups has similarly been documented in many other studies [8,9].

**Figure 1:**
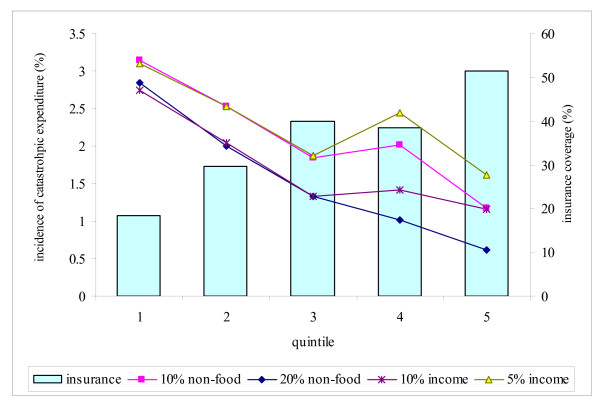
**NHIS Status and Incidence of Catastrophic OOP Expenditures among Wealth Quintiles **Note: underlying figures are from the data used in this study, which consist of 11,617 individuals surveyed in 2007 in two rural districts Nkoranza and Offinso. Statistics are adjusted using sampling weights.

The socioeconomic gradient observation is corroborated in Figure 1, which plots insurance coverage and incidences of high and catastrophic payments against wealth quintiles. As revealed, there is a clear upward trend in insurance coverage, with the level among richest quintile being more than 2.5 times higher than that among the poorest quintile (50 percent versus 20 percent, respectively). Correspondingly, there is also a clear downward pattern of high and catastrophic expenditure, with poor people having much higher incidence compared with the rich on all measures. Besides the clear concern about equity in coverage and financial protection, the patterns in Figure 1 suggest some relationship between insurance coverage and large OOP payment, which we will test formally in the subsection that follows.

### The Effect of NHIS on OOP Payment

Table 4 presents the first set of estimation results assessing the effects of NHIS on the absolute amount of OOP payment. Model 1 controls for self-reported chronic health conditions and health status while model 2 omits these variables. Both models include individual and household characteristics described in table 3, namely the person's age, age squared, gender, ethnic Akan, if s/he is a member of a solidarity scheme, household size, the household head's gender, education, and occupation, as well as household's assets and living conditions. For clarity of presentation, the effects of these covariates are not shown in the table because they are not of primary interest. The key figures of interest are those for "has HI from NHIS," which show the marginal effects of insurance on OOP expenditure. As explained earlier, these marginal effects, estimated from the two-part model, combine insurance effects on the probability of having any OOP payment and the absolute amount given that a payment is made. The robust standard errors, adjusted for sampling weight and clustered at the municipality level, were estimated by boostrapping with 200 replications.

**Table 4:** The Effect of NHIS on Average OOP Expenditure (Cedi)

	OOP on health services (Two-part model)	
	(1)	(2)
Has HI from NHIS	-33,821	-30,094
	(20,379)*	(20,157)
Chronic health condition	40,605	---
	(38,229)	
Bad health (self-assessed)	125,223	---
	(90,323)	
Offinso district	12,946	13,032
	(5,205)**	(4,362)***
Individual and household characteristics	Yes	Yes
Assets and living conditions	Yes	Yes
N	11,617	11,617

*Note*: Cedi is old Ghana Cedi, 2007 current value; exchange rate: 1 USD = 9,302 Cedi (2007)

Individual and household characteristics include gende, ethnic Akan, age, age squared, if a member in solidarity scheme, household size, and gender, education, and occupation of the household head.

Assets include radio, TV, fridge, telephone, bicycle, motorbike, and car. Household living conditions include whether there is electricity, floor type, type of water, and fuel used for cooking; owning a house, having farmland, and number of rooms in the house.

Figures presented are marginal effects of NHIS and their robust standard errors (in parentheses), adjusted for sampling weight and clustering effect at the municipality level. The standard errors are estimated by boostrapping with 200 replications.

*** p < 0.01, ** p < 0.05, * p < 0.1

Both models 1 and 2 show that the insurance effect is in the expected direction, i.e. insurance has been shown to reduce OOP among its members. The marginal effects of insurance are smaller (in absolute value) when negative health status indicators are not controlled for (model 2 compared to model 1), suggesting that the derived estimates could be on the lower bound if adverse selection due to poor health status has not been fully controlled for. If there remain omitted variables that drive adverse selection in the same way as health status (e.g., residual wealth status), the true insurance effect will be larger than that observed in models 1 and 2.

It is noteworthy that in model 1, insurance effect is only marginally significant (at 10 percent level) and rather small (33,821 old Cedi). To examine its magnitude relative to household capacity to pay, we compare that amount with the average figure on household non-food consumption expenditure from Brong Ahafo region, where Nkoranza is located. We take the household non-food consumption expenditure per capita for Brong Ahafo reported in the Ghana Living Standard Survey 2005-2006 (Tables 9.2 and 9.5) [28] and adjust it for inflation and real growth to obtain the comparable figure for 2007, which is 2,701,300 Cedi. Thus, an effect of 33,821 Cedi would be equal to 1.25 percent of non-food consumption expenditure, which is not substantial. Again, it is important to keep in mind the large confidence interval of this estimate (0.24 percent-2.75 percent of non-food consumption expenditure).

### The Effect of NHIS on Catastrophic Health Payment

Table 5 shows the estimation results for the various catastrophic OOP payment measures. All estimations control for health status and usual individual and household characteristics. A clear and consistent pattern emerges across all measures: having NHIS significantly reduced the probability of catastrophic OOP payment on health services. The estimated reduction ranges from 0.5 percentage point (for expenditure of at least 20 percent of non-food expenditure) to 1 percentage point (for expenditure of at least 5 percent of income). In relative terms, a reduction of 1 percentage point would be 67 percent compared with the mean of the insured and 36 percent compared with the mean of the uninsured (Table 3). Consistent with the finding on OOP expenditure, males have a significantly lower incidence of catastrophic payment. Interestingly, while Offinso is less poor than Nkoranza, Offinso residents were more likely to incur catastrophic payment. Because Offinso has more hospitals, more private and mission clinics, and higher insurance premiums and registration fees, it is possible that the price of services is also higher in Offinso, which explains higher incidence of catastrophic payment. However, further data and investigation are needed to form a rigorous explanation of the difference between the two study districts.

**Table 5:** The Effect of NHIS on Various Indicators of Catastrophic OOP Expenditure on Health

Thresholds	5% income	10% non-food expenditure	10% income	20% non-food expenditure
	(1)	(2)	(3)	(4)
Has HI from NHIS	-0.010	-0.007	-0.006	-0.005
	(0.004)***	(0.003)**	(0.003)*	(0.002)**
Chronic health condition	0.015	0.014	0.016	0.014
	(0.017)	(0.016)	(0.016)	(0.013)
Bad health (self-assessed)	0.048	0.042	0.041	0.033
	(0.031)	(0.028)	(0.028)	(0.023)
Offinso district	0.013	0.012	0.011	0.010
	(0.003)***	(0.003)***	(0.002)***	(0.002)***
Other individual and household characteristics	Yes	Yes	Yes	Yes
Assets	Yes	Yes	Yes	Yes
N	11,617	11,617	11,617	11.617

*Note*:Individual and household characteristics include gender, ethnic Akan, age, age squared, if a member in solidarity scheme, household size, and gender, education, and occupation of the household head.

Assets include radio, TV, fridge, telephone, bicycle, motorbike, and car. Household living conditions include whether there is electricity, floor type, type of water, and fuel used for cooking; owning a house, having farmland, and number of rooms in the house.

Figures presented are marginal effects of NHIS and their robust standard errors (in parentheses), adjusted for sampling weight and clustering effect at the municipality level.

*** p < 0.01, ** p < 0.05, * p < 0.1

A question of interest is whether the effect of insurance works differently for the poor versus non-poor. We examine this by performing separate estimations for the poorest 20 percent versus the rest of the sample. The results, presented in Table 6, are telling. The effects of insurance are large and strongly significant in all measures among the poor, while marginally or non-significant among the rest of the sample. In fact, most of the average effect observed in Table 4 actually comes from effect among the poor. For the rest of the population, the effects have the expected direction of effects, but are too small to yield any statistical significance.

**Table 6:** The Differential Effect of NHIS on the Incidence of Catastrophic OOP Expenditure on Health by Wealth Status

Indicators	Poorest quintile N = 1,762	Rest of population N = 9,855
Exceeds 5% of income	-0.016 (0.005)***	-0.007 (0.004)
Exceeds 10% of non-food expenditure	-0.017 (0.005)***	-0.004 (0.004)
Exceeds 10% of income	-0.013 (0.005)**	-0.004 (0.003)
Exceeds 20% of non-food expenditure	-0.014 (0.005)***	- 0.003 (0.002)*

*Note*:Figures are obtained from separate estimations among the poorest and the rest of the sample. The models control for self-reported chronic health conditions and bad health status, all individual and household characteristics, assets and housing conditions, and district dummy. Figures presented are marginal effects of NHIS and their robust standard errors (in parentheses), adjusted for sampling weight and clustering effect at the municipality level.

*** p < 0.01, ** p < 0.05, * p < 0.1

## Discussion

This study finds evidence that the NHIS in Nkoranza and Offinso has helped reducing the incidence of catastrophic OOP expenditure among its members. Depending on the indicators, the probabilities of catastrophic expenditure decreased by 0.5 to 1 percentage point among NHIS members, a reduction of 36 percent to 67 percent of the sample means. In terms of the absolute amount of OOP expenditure, insurance's marginal effect is small and only significant at 10 percent. These seemingly contradictory patterns can be explained by the higher volume of service consumed by insured patients, as shown in the existing literature [9,20], and by the fact that OOP payment did take place at the point of service even for the insured. Still, insurance has served its function as a safety net mechanism, reducing the probability that households have to forego other subsistent needs for health care.

The protective effect of insurance against catastrophic expenditure is particularly strong among the poor, who are typically more vulnerable to health shocks than the rest of the population. This finding supports the Ghanaian government's decision to exempt premium for the indigenous. In fact, given that insurance coverage is substantially lower among poor people, further efforts are needed to enroll this vulnerable population. For example, the criteria for being considered as "indigent," and hence exempted from premiums, are currently too strict [9]. They exclude many people who are poor but not poor enough to enjoy free insurance. The government may decide to relax the criteria and implement more promotion activities in order to enroll a larger pool of the poor people.

A closer examination of the results of this study reveals a more complicated picture. Despites NHIS's generous benefit packages, its members still incurred an OOP payment that equals 72 percent of that incurred by nonmembers. A nontrivial portion of this amount is spent on items that should be covered by insurance, such as drugs, tests, antenatal care, and hospitalization. These findings raise a number of questions on the implementation aspects of insurance which are highly related to the early experience with NHIS reported in the existing literature [18]. For example, is there a tendency for providers to direct insured patients to services, drugs, and tests that are not covered by insurance, so that they can collect more revenue? Is the quality of care comparable in the services rendered to the insured and uninsured patients? Is the procedure cumbersome enough to discourage the patients from obtaining insured services? What are the possible advantages of the unaccredited facilities, such as convenience and quality, that attract insured patients to go there knowing that they will have to pay out of pocket?

As noted earlier, because OOP payment is the net effect of the change in unit price and volume of services utilized, the presence of OOP payment among the insured is not necessarily bad. It has been shown that NHIS has brought about a significant increase in health care demand among its members [8,9]. Insured people thus may be willing to pay to obtain extra services and tests that they may not have had without insurance. This "access value" of insurance has been documented elsewhere and can well be the case with Ghana [32].

The current analysis ultimately has some limitations. We have not been able to address fully the selectivity of insurance. Although we can control for chronic conditions and self-assessed bad health status, there remain potentially omitted variables that bias these results. As such, the documented association may not represent the true magnitude of the NHIS effects on the outcomes of interest. Most likely, we believe that bias, if exists, will make the results conservative and the true magnitude of the effects may be higher than what is documented here. The second drawback results from the lack of household income and expenditure data in our sample, which renders the estimates imprecise. The low percentage of the studied population having had any positive health expenditure and the skewness of expenditure data have always been a challenge in health service research. Finally, our results only pertain to two out of 138 districts of Ghana, and hence cannot be generalized to the country as a whole.

Future studies can build on this research in many respects. For example, to assess more systematically the equity aspect of the Ghana NHIS, benefit incidence analysis would be suitable to document how much people with different wealth status benefit from the NHIS. With better data, one could explore the effects of differentiating the threshold z for catastrophic health expenditure across income levels. It would be useful to quantify the degree of adverse selection as it will affect the financial viability of the insurance fund, especially when coverage is still low. Moral hazard from members could eventually become a problem with the NHIS due to the generous benefit package and lack of requirements for coinsurance. Finally, some in-depth case studies will be useful to shed light on the operational aspects of NHIS and on quality of care for its members.

## Conclusion

Limitations notwithstanding, this study makes a potentially valuable contribution to the evaluation of the Ghana NHIS in particular and impact evaluation of health insurance in general. It shows that with strong commitment to social health insurance , a poor country can achieve financial protection against health care cost for its population, in particular the most vulnerable group. Another lesson learnt from Ghana is that instituting insurance by itself is not adequate to remove fully the out-of-pocket payment for health care. Insured patients are still required to pay for items that should be covered by insurance and for informal care. Without proper regulation and incentives for the supply side to improve quality and availability of services, insurance cannot be an attractive product. This in turn will be a hindering factor in coverage expansion and ultimately will affect the prospect of achieving universal coverage.

Ghana's experience is applicable to many developing countries, especially the countries in sub-Saharan Africa. The context for social health insurance is rather similar. Most countries in the sub-Saharan Africa in the 1980s and 1990s underwent health financing reform where user fees in the public facilities were instituted and different forms of the MHOs were piloted. Social health insurance is being considered as a way to go for alleviating financial burden and improve access to health services. The results on protection effect against catastrophic OOP expenditure are encouraging for many low income countries, which are on the way to develop and expand their social health insurance.

## Appendix

Proof of the sign of bias in linear models

Suppose there is only one variable in Z, called z, that is systematically associated with HI status and Y, then the true relationship between Y, HI, and z can by modeled as:

(1) $$\text{Y}=\alpha_{\text{1}}+\beta_{\text{1}}\text{HI}+\gamma_{\text{1}}\text{z}+\varepsilon_{\text{1}}$$

Not being able to observe z, we could wrongly model the relationship as:

(2) $$\text{Y}=\alpha_{\text{2}}+\beta_{\text{2}}\text{HI}+\varepsilon_{\text{2}}$$

Because z is systematically related to HI, the relationship between them can be specified as:

(3) $$\text{z}=\alpha_{\text{3}}+\delta_{\text{3}}\text{HI}+\varepsilon_{\text{3}}$$

Substitute (3) in (1), we have:

$$\text{Y}=(\alpha_{\text{1}}+\gamma_{\text{1}}\alpha_{\text{3}})\;+\;(\beta_{\text{1}}+\gamma_{\text{1}}\delta_{\text{3}})\text{HI}+\;(\gamma_{\text{1}}\varepsilon_{\text{3}}+\varepsilon_{\text{1}})$$

Thus, without controlling for z, the estimated effect of HI is (β_1_+ γ_1_δ_3_), while the true underlying effect is β_1_. γ_1_δ_3 _is the bias caused by omitting z. If z is something that is positively correlated with both insurance and health expenditure (such as poor underlying health), both γ_1 _and δ_3 _will be positive and the bias will be positive. Note that the bias is positive as well if z is negatively correlated to both insurance and health expenditure (such as low wealth status). Because the expected insurance effect on Y in this study is negative (a reduction in OOP), a positive bias will result in an estimation that is smaller than the true effect in absolute value.

## Competing interests

The authors declare that they have no competing interests.

## Authors' contributions

HTHN developed the analytical approach, performed the statistical analysis, and drafted the manuscript. YR and HW contributed to the drafting of the manuscript. All authors have read and approved the final manuscript.
